# Information Technology Measurement and Testing Activities at NIST

**DOI:** 10.6028/jres.106.013

**Published:** 2001-02-01

**Authors:** Michael D. Hogan, Lisa J. Carnahan, Robert J. Carpenter, David W. Flater, James E. Fowler, Simon P. Frechette, Martha M. Gray, L. Arnold Johnson, R. Michael McCabe, Douglas Montgomery, Shirley M. Radack, Robert Rosenthal, Craig M. Shakarji

**Affiliations:** National Institute of Standards and Technology, Gaithersburg, MD 20899-0001

**Keywords:** conformance, hardware, information technology, interoperability, measurement, performance, reference data, software, testing

## Abstract

Our high technology society continues to rely more and more upon sophisticated measurements, technical standards, and associated testing activities. This was true for the industrial society of the 20th century and remains true for the information society of the 21st century. Over the last half of the 20th century, information technology (IT) has been a powerful agent of change in almost every sector of the economy. The complexity and rapidly changing nature of IT have presented unique technical challenges to the National Institute of Standards and Technology (NIST) and to the scientific measurement community in developing a sound measurement and testing infrastructure for IT. This measurement and testing infrastructure for the important non-physical and non-chemical properties associated with complex IT systems is still in an early stage of development. This paper explains key terms and concepts of IT metrology, briefly reviews the history of the National Bureau of Standards/National Institute of Standards and Technology (NBS/NIST) in the field of IT, and reviews NIST’s current capabilities and work in measurement and testing for IT. It concludes with a look at what is likely to occur in the field of IT over the next ten years and what metrology roles NIST is likely to play.

## 1. Introduction

Our high technology society and economy rely more and more upon sophisticated measurements, technical standards, and associated testing activities. This was true for the industrial society of the 20th century and remains true for the information society of the 21st century. The contributions of NBS/NIST to the development of measurements for physical and chemical properties have been widely accepted from the earliest days of the establishment of the institution. NBS/NIST measurement work is based on recognized scientific principles and techniques, and, over the years, it has supported the evolving needs of industries such as steel, machine tools, automobiles, and chemical processing for accurate and precise measurements.

Over the last half of the 20th century, Information Technology (IT) became a powerful agent of change in almost every sector of the economy. Today, IT continues to play a significant role in improving products and services, making people more productive, and supporting a strong economy. The complexity and rapidly changing nature of information technology have presented unique technical challenges to NIST and to the scientific measurement community in developing a sound measurement and testing infrastructure. This measurement and testing infrastructure for the important non-physical and non-chemical properties associated with complex information technology systems is still in an early stage of development.

The *American National Standard Dictionary of Information Technology* (ANSDIT) [1] defines the term, *information technology*, as follows:

The art and applied sciences that deal with data and information. Examples are capture, representation, processing, security, transfer, interchange, presentation, management, organization, storage, and retrieval of data and information.

IT systems, which are typically a blend of hardware and software, can be characterized as becoming more digital. The hardware can be described as becoming ever more complex and difficult to manufacture. The software can be characterized as becoming more complex and difficult to develop while easy to replicate. IT systems, including computers, computer networks, telephones, telephone networks, televisions, and cable networks, are ubiquitous, impacting all industries (manufacturing, health care, education, etc.), and all aspects of the economy. The ongoing challenge is to develop IT measurement and testing methods that will ensure that these complex IT systems are usable, scalable, secure, and interoperable. With the exception of the measurement of time, measurement and testing of attributes of digital IT systems do not rely upon measuring with the International System of Units (SI). Rather IT measurement involves ascertaining the correctness of information or data when stored, processed, transferred, displayed, managed, organized, retrieved, etc.

Definitions for several terms already mentioned in the context of IT will help to further conceptualize the scope of this paper.

The *American National Standard Dictionary of Information Technology* (ANSDIT) defines the terms, *data* and *information*, as follows:

*data* (ANSDIT)

Any representation subject to interpretation (such as through analysis or pattern matching) or to which meaning may be assigned, such as by applying social conventions or special agreed upon codes. Data can be processed by humans or by automatic means.

*information* (ANSDIT)

(1) The meaning that is currently assigned to data by means of the conventions applied to these data. (2) In information processing, any fact, concept, or meaning derived from data and associated context or selected from knowledge.

**Fig. 1:**
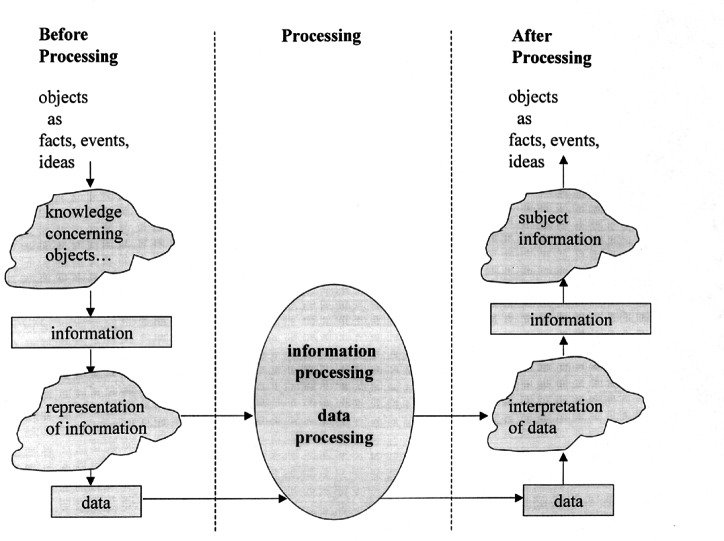
Concepts of data and information processing.

Figure 1, also from the *American National Standard Dictionary of Information Technology* (ANSDIT), illustrates the concepts of *data* and *information* processing.

The *American National Standard Dictionary of Information Technology* (ANSDIT) also gives the following definitions for *hardware* and *software*:

*hardware* (ANSDIT)

Any physical component capable of data processing, for example, computers, peripheral equipment.

*software* (ANSDIT)

All or part of the programs, procedures, rules, and associated documentation of a data processing system or an information processing system. Software is an intellectual creation that is independent of the medium on which it is recorded.

From these definitions, it is apparent that hardware has both physical and other data processing attributes. In contrast, software is composed of intellectual or logical attributes, and, with the exception of time, physical attributes are not essential.

It is interesting to note that the terms, *measurement* and *testing*, are not defined in the same vocabulary document. The *International Vocabulary of Basic and General Terms in Metrology* (the VIM) [2] defines *measurement* but not *test* or *testing*. *ISO/IEC Guide 2: 1996, Standardization and related activities—General vocabulary* [3] defines *test* and *testing* but not *measurement*.

*measurement* (VIM)

Set of operations having the object of determining a value of a quantity.

*test* (ISO/IEC Guide 2)

Technical operation that consists of the determination of one or more characteristics of a given product, process or service according to a specified procedure.

*testing* (ISO/IEC Guide 2)

Action of carrying out one or more tests.

Measurement and testing appear to be defined so that these terms are either conceptually equivalent or, at least, closely related. Therefore, the terms, measurement and testing, are often combined in this paper because of their rough equivalence or overlap. A distinction between these terms is also sometimes made by considering testing to be a measurement or measurements together with a comparison to a specification.

## 2. Historical Development of IT Measurement and Testing Activities at NBS/NIST

NBS/NIST activities that support the development and measurement of digital systems have their origins in the work that the organization did during World War II. In the late 1940s NBS was invited to be the technical consultant for the procurement of digital computers by the Bureau of the Census, the Office of Naval Research, and the Department of the Army. When the computers that were ordered from commercial sources were not ready in time to carry out the needed functions, NBS made plans to build a small “interim” computer that would provide the needed computing capabilities. This project to build a computer was adopted partly because of the delay in delivery of the commercial computers and partly to enable NBS to gain experience in machine construction and design [4]. NBS was in a position to take on this task because it had developed significant expertise in electronics during World War II, particularly in the production of electronic components and development of printed circuits.

NBS began construction of the Standards Electronic Automatic Computer (SEAC) in 1948 and completed the project in 1950. SEAC was later renamed the Standards Eastern Automatic Computer when a companion computer was built in California in 1956 and named the Standards Western Automatic Computer (SWAC). SEAC was the only stored program machine in the United States, and was the fastest such machine in the world at the time. It had 512 words of memory (44 bits per word), a cycle time of one megahertz, and punched paper tape input and output. SWAC was a parallel machine with magnetic drum memory. SEAC was used to work on many problems including solving partial differential equations by Monte Carlo methods, generating optimum sampling plans for the Census Bureau, calculating transient stresses on aircraft structures, and developing accounting procedures for the Social Security Administration. SEAC was retired in 1964, and its parts are at the Smithsonian Institution.

After the development of SEAC and SWAC by NBS in the late 1940s and 1950s, computers became commercial realities. In the 1950s, the computer industry began to grow despite often quoted pessimistic estimates of the number of computers that would be needed in the world. As computers were adopted by the public and private sectors, Federal agencies no longer needed a government agency to develop computers for them, but needed instead a base of government competency in computer hardware and software technology to assist them in managing and using the new electronic devices.

The technical expertise that NBS had gained in computer technology at that time was recognized when Public Law 89-306 (79Stat.1127) was enacted on October 30, 1965, as an amendment to Title I of the Federal Property and Administrative Services Act of 1949 (63Stat.377). The purpose of this legislation, known as the Brooks Act, was to “provide for the economic and efficient purchase, lease, maintenance, operation, and utilization of automatic data processing equipment by Federal departments and agencies.”

Under the Brooks Act, three agencies were given central management responsibilities for automatic data processing (ADP) activities. The General Services Administration (GSA) was directed to coordinate and provide for the economic and efficient purchase, lease, and maintenance of ADP equipment by Federal agencies. The Secretary of Commerce was authorized to provide scientific and technological advisory services to Federal agencies and to GSA, to make recommendations to the President relating to the establishment of uniform Federal ADP standards, and to undertake necessary research in computer sciences and technology. The Office of Management and Budget (OMB) was assigned responsibility for exercising fiscal and policy control over the executive functions assigned to GSA and the Secretary of Commerce.

The authorities assigned to the Secretary of Commerce for standards development, technical assistance and research were subsequently delegated to the National Bureau of Standards. Executive Order 11717, dated May 9, 1973, transferred to the Secretary of Commerce all functions being performed by the Office of Management and Budget relating to the establishment of Government-wide automatic data processing (ADP) standards, including the function of approving standards on behalf of the President.

As the government continued to expand its use of computers in the 1960s, users experienced problems of incompatibilities among hardware, software, and computer-generated data. Standards were recognized as the principal tool for solving this problem. To carry out its responsibilities under the Brooks Act, NBS established the Federal Information Processing Standards (FIPS) program to develop standards for data, programs, and components, data communications, computer performance, applications and data, personnel and environment and acquisition and reassignment of ADP products. The Federal government supported the development of standards as an effective way to facilitate the interchange and sharing of data, programs, and equipment by Federal agencies. This focus on standards helped to increase awareness by government and industry of the need for compatibility and more effective utilization of ADP products and services.

During the 1970s and 1980s NBS/NIST worked with private sector standards organizations to develop technical standards that were used by both public and private sectors. At the time, NBS was one of few government organizations with the technical expertise needed to support the development of both voluntary industry standards and government-developed standards that met unique government needs. These standards were developed by NBS, both in conjunction with standards organizations and with other government agencies. Standards that NBS thought suitable for use by other government agencies went through an open, public review process before they were submitted to the Secretary of Commerce for approval. Approved standards were issued as Federal Information Processing Standards (FIPS) and specified for government-wide use.

A major augmentation of NBS’s responsibilities to develop standards and guidelines for Federal computer systems occurred in 1987 when the Computer Security Act was passed. Managers at all levels in Federal organizations were confronting difficult challenges in protecting the information processed by their organizations’ computer systems. IT was becoming an essential component of many government activities; many organizations and individuals were communicating and starting to do business via computer networks. These systems and networks were vulnerable to threats such as intruders, hardware and software failures, outages, and viruses that cost users millions of dollars in lost time and productivity. Federal managers needed ways to assure that the confidentiality, integrity, reliability, and availability of their information resources was safeguarded.

Under the Computer Security Act of 1987, NBS was designated as responsible for developing technical, management, physical security, and administrative standards and guidelines for the cost-effective security and privacy of sensitive unclassified information processed in Federal computers. NBS/NIST worked with the private sector and with government agencies to address the common needs of both groups of users for improved security. NBS/NIST carried out developmental projects in security awareness, user training, authentication technology, contingency planning, network security, and encryption, and issued recommendations of good practices, standards, and guidance.

In 1996, the Brooks Act was replaced by the Information Technology Management Reform Act (Public Law 104-106). This legislation reenacted NIST’s responsibilities to develop standards and guidelines for Federal computer systems. The Secretary of Commerce was directly authorized to approved standards and guidelines developed by NIST. Between 1968 and 1996, about 190 of these standards and guidelines were issued as Federal Information Processing Standards (FIPS) for use government-wide. The FIPS series included standards for security, interoperability, and interchange of data, many of which adopted for government-wide use the voluntary industry standards, that NIST/NBS staff members helped to develop.

In 1996, the policy of issuing FIPS that adopted voluntary industry standards was modified as NIST changed its focus to concentrate on developing tests, measurements, proofs of concept, reference data and other technical tools to support the development of pivotal, forward-looking technology. NIST determined that FIPS were to be developed when there were compelling Federal government requirements such as for security and interoperability and there were no acceptable industry standards or solutions.

This policy was in accordance with the National Technology Transfer and Advancement Act of 1995 (Public Law 104-113) and Administration policies, which supported the development of voluntary industry standards both nationally and internationally as the preferred source of standards to be used by the Federal government. As a long time supporter of voluntary industry standards, the Federal government prefers to use these industry standards, rather than creating its own standards. As a result, the government is able to rely upon the private sector to supply it with goods and services. NIST continues to collaborate with national and international standards committees, users, industry groups, consortia, and research and trade organizations, to get needed standards developed, but focuses mainly on issues of information security for new and revised FIPS.

The need for tests, measurements, proofs of concept, reference data and other technical tools to support the development of new technology became more critical as information technology systems became more complex. NIST’s capabilities to measure and test the attributes of digital IT systems had their beginnings in the expertise gained with the building of the digital electronic computer, SEAC, in the 1940s and 1950s and with the development of Federal Information Processing Standards (FIPS) in the 1960s. Some of the NBS scientists and engineers who worked on SEAC subsequently became involved in measurements for magnetic computer storage media. Many of the NBS staff developing FIPS also developed testing methodologies and tests for the software that implemented the complex technical specifications in many of the FIPS.

Four examples of past projects that helped to advance testing and measurements of IT systems include measurements of the electrical and magnetic properties of computer storage media, development of conformance tests for programming languages, development of interoperability tests for local area networks, and performance measurements for parallel processors.

### 2.1 Measurements of the Electrical and Magnetic Properties of Computer Storage Media

The ability to interchange data stored on digital magnetic media was a new and important operational requirement of computer systems of the 1960s to 1970s. However, interchange between tapes produced by different manufacturers was not easily achieved because of differences in dynamic signal amplitude response. In testing tapes, NBS scientists found that there was a dynamic signal amplitude response spread of almost 50 % on a comprehensive sample of manufacturers’ production control tapes. The off-the-shelf tapes produced by these manufacturers would have had an even greater spread in signal values, making it difficult to interchange data from tapes produced by different manufacturers.

The NBS Magnetic Media Storage Group addressed this problem by developing standard reference materials and calibration services for computer storage media. This enabled manufacturers of different types of storage media to design their products on the basis of a comparison to a known and generally accepted standard reference tape or disk.

The standard reference materials and calibration services developed by NBS were used by government and industry media testing laboratories and by manufacturers of the media and associated data storage equipment. NBS’s work led to improvements in product quality control and more efficient data processing operations in government and industry. Tests made after the reference materials and services had been available for only a short time showed a marked improvement in the uniformity of values for dynamic signal amplitude response. This work was unique since there were no alternative industrial expertise and sources for these materials and services in the 1970s and 1980s.

### 2.2 Conformance Testing for Programming Languages

Variations in the compilers that the Federal government acquired for the Common Business oriented Language (COBOL) programming language led to the need for better quality control, and to NBS activities to develop conformance tests for programming languages. Compilers often implemented the features of the language that the implementers chose or more importantly implemented features incorrectly, resulting in difficulty transferring programs among different compilers. The Department of Defense (DoD) started the development of tests for the COBOL [5] and Fortran programming languages. NBS also developed COBOL and Fortran tests that were added to enhance the earlier DoD tests. Later this work was transferred to NBS where test development was continued [6] [7]. This early work in programming language conformance test development set the stage for conformance test development by private sector and international companies in C and COBOL respectively with technical guidance from NBS/NIST and standards committees. In addition, tests were developed for other languages such as Beginner’s All-Purpose Symbolic Instruction Code (BASIC) [8], Ada, and Structured Query Language (SQL) [9]. The tests captured the technical description of standards specifications, and were used to measure whether products implemented the specifications correctly.

The development of conformance tests complemented NBS/NIST participation in the development of industry technical standards, and helped support the implementation and use of these standards. At the time that the programming language standards were under development, NBS/NIST staff members had the scarce technical skills and experience that were needed to sustain the standards work in national and international standards organizations. With the support of the members of the standards committees, NBS/NIST operated testing services that applied the test suites to products. Certificates of conformance were issued as appropriate. Experience in using the test suites helped to make the writing of standards more precise because the specifications became more definitive and contained fewer areas that were left to definition by the implementers.

The primary purpose of these early IT measurement efforts was to help federal agencies buy quality products, but the efforts also helped industry by advancing the application of quality processes to compiler development. The use of conformance tests in Government operated testing services had a significant impact on the quality of products and their compliance to FIPS standards [10]. By providing neutral ways to measure products, the conformance tests helped buyers and users to select standards-conforming products. At the same time, the tests helped developers find errors in their implementations and thus to improve the quality of their programming language products.

### 2.3 Interoperability Testing for Local Area Networks

During the 1980s, NBS staff members played a pivotal role in the development of local area networking technology. During the early and mid 1970s experiments were conducted on the Advanced Research Projects Agency (ARPA) Network, the predecessor to today’s Internet. The NBS Experimental Computer Facility hosted the 6th ARPA Net node—a terminal interface message processor. By the end of the 1970s the geographically distributed resource sharing ARPA Network computers sometimes stopped functioning, but the technology was demonstrable. In contrast to these wide area networks, the new challenge was to understanding how to provide rich connectivity for a campus, office complex or factory floor where all the resources including the physical cable were locally owned, operated and administered. This new approach was called a local area network or LAN.

In May of 1979 NBS and the Mitre Corporation teamed up to cosponsor the first Local Area Communications Network Symposium [11]. While significant advances in broadband cable LAN technology were reported, the symposium attendees were particularly enthusiastic about the emergence of a new technology called Carrier Sense Multiple Access with Collision Detection or CSMA/CD. Digital Equipment Corporation, Intel Corporation and the Xerox Corporation produced a specification for a three megabit per second coaxial cable based CSMA/CD system to meet the needs of the “office automation” community. A report was given on the NBS work on a campus-wide CSMA/CD network called NBSNET. From those experiences and with the help of many others, NBS started a voluntary international standards effort known as the Institute of Electrical and Electronics Engineers (IEEE) Project 802. Building on the good ideas of over 100 experts from companies all over the world, a family of LAN standards emerged.

NBS built a LAN protocol testing laboratory to study and measure implementations of these new ideas in an attempt to help industry better understand interoperability and performance issues. Experiences with NBSNET and other commercial implementations of CSMA/CD led to the publication of Federal Information Processing Standard 107 [12] to foster interoperability of the myriad products offered to solve office automation problems.

The manufacturing automation community and the process control industry needed a different LAN standard and focused on Broadband Token Passing Bus. This technology eventually became another member of the Project 802 family of standards.

At the request of industry, NBS established a Manufacturing Automation Protocol Testing Laboratory. The goal was to help end users and original equipment manufactures link automated machinery and smaller mini and micro systems and controllers from different manufacturers, and enable the systems to work together on the factory floor. Users wanted cost effective, off-the-shelf solutions for distributed computer applications. To demonstrate Token Passing Bus interoperability, a test methodology was designed by NBS scientists and engineers and tested by equipment vendors working closely with NBS.

This work resulted in a deeper understanding of the interoperability issues affecting Broadband Token Passing Bus protocols. By demonstrating specific interoperability problems in repeatable laboratory experiments using state-of-the-art measurements, NBS helped to achieve evolutionary changes in the emerging voluntary standards that ultimately lead to better network products.

### 2.4 Performance Measurement of Parallel Processors

NBS/NIST developed Multikron^®^, a series of Very Large Scale Integration (VLSI) instrumentation chips and interface boards that capture performance data to promote both high-performance computing and flexible scalable systems. The system measures the performance of parallel processors and workstations on high-speed networks by recording events triggered either by software memory writes or the transition of hardware signals. The chips can either timestamp the captured data and send it over a collection network or use it to control counters and clocks aboard the chip. The resulting accurate measurements permit researchers to understand the source of performance bottlenecks and therefore learn how to scale their system designs upwards without significantly perturbing the system under measurement.

Development of concepts and hardware to enable low perturbation and high accuracy performance measurement of computer systems and networks began in the mid-1980s at NBS. It was recognized that traditional approaches using software capture of a computer’s time clock were inadequate: the many software instructions entailed high overhead; computers usually had poor resolution from their standard clocks. A substantial improvement in time resolution and a great reduction in instrumentation perturbation would occur if timestamps and other data could be captured by the initiation of a simple “write” instruction to a memory-mapped device. Any timing clock and its reading were the responsibility of the special hardware—the only system perturbation was the “write” instruction that signaled the event. This approach was one of two initially pursued in the course of developing the NBS/NIST TRAce Measurement System (TRAMS) and the later MultiKron^®^ devices. Early in the effort, inexpensive fabrication of small custom integrated circuits became available through the Defense Advanced Research Projects Agency (DARPA) Metal Oxide Semiconductor Implementation System (MOSIS) service. This allowed much of the instrumentation hardware to be contained in custom integrated circuits designed expressly by NIST.

A second, parallel measurement approach within the project consisted of a NIST-developed printed circuit board which contained a pattern matcher, usually matching processor address lines, and a 36 bit timestamp counter which was incremented at a 10 MHz rate [13]. Events were defined by the pattern matcher and caused no perturbation in the computer’s sequence of instructions. When a pattern match was triggered, it caused transfer of the current value of the timestamp counter and other identifying data to a first-in, first-out (FIFO) data memory. All this was outside of the measured system. Event data was time-stamped to a 100 ns resolution. The event trigger could also cause the capture of data in one or more NIST custom instrumentation chips, which each contained four counters that could accumulate information on the utilization of various portions of the system under test.

While passive, pattern-matching triggering avoided introducing program perturbations, a need was recognized for considerably more trigger points than were practical in such a matching system. Furthermore, existing key pattern trigger features, such as virtual addresses, would soon be unavailable when embedded within a microprocessor chip. Events would be hard to define and to observe, so for each event, it was decided to accept the perturbation of the single computer “write” instruction. Explicit “write” instructions in the code under test would define measurement events. Data associated with each “write” instruction could identify which active software trigger caused the data capture. In committing to this view, the number of available defined events became far larger than with passive pattern triggering. Furthermore, events corresponded directly to details within the code being investigated. Programmers liked this feature, which was necessary for unambiguous measurement values.

Fabrication improved rapidly. Larger-scale custom integrated circuits were practical for small-scale experimenters, so essentially all of the second-generation measurement-assist hardware became contained in a single MultiKron^®^ chip [14]. MultiKron^®^ has a 56 bit times-tamp counter and 16 performance counters (32 bits per counter). All the counters can be incremented at frequencies up to 50 MHz, for a 20 ns resolution. MultiKron^®^ chips have been attached to each processor in multiprocessor computer systems, with all the MultiKrons^®^ driven by the same clock frequency and with their timestamps synchronized. This allows a time-correlated comparison of the actions on the various processors in the system. In addition, hardware has been developed that uses signals from the Global Positioning System (GPS) satellites to allow synchronization of MultiKron^®^ chips at locations separated by thousands of kilometers. This capability is important for measurement of computer and communication networks. [15, 16].

## 3. Present IT Measurement and Testing Activities at NIST

In 1996, NIST management established an inter-laboratory study group to discuss and review the status, needs, and challenges of providing measurements for information technology (IT). The study group, composed of representatives from the Manufacturing Engineering Laboratory (MEL), the Information Technology Laboratory (ITL), and Technology Services (TS), had a wide spectrum of experiences and perspectives on testing and measuring physical and IT quantities. The group developed a white paper (NISTIR 6025), *Metrology for Information Technology* [17], which became the starting point for discussions with industry and with the scientific measurement community to address the overall subject of metrology for IT. NISTIR 6025 suggested a conceptual basis for metrology, and reviewed IT testing methods, the status of IT metrology, and opportunities for advancing IT metrology.

Metrology is defined in the *International Vocabulary of Basic and General Terms in Metrology* (the VIM) as the science of measurement. The VIM notes that metrology includes all aspects both theoretical and practical with reference to measurements, whatever their uncertainty, and in whatever fields of science or technology they occur.

NISTIR 6025 concluded that IT metrology is a valid branch of metrology. IT metrology differs from physical metrology in several ways including: the SI dimensioning system is not as relevant; less analytical methods exist to quantify uncertainty: and the area is relatively new compared to physical metrology. As a result, IT metrology has its own unique set of challenges, opportunities, and priorities.

IT system hardware can be very complex but relatively easy to measure because it relies upon mature and sophisticated physical and chemical measurement science. Software, on the other hand, is increasingly complex and difficult to measure due to the limitations of present software metrics [18].

The concept of calibration is well understood in the physical metrology community. Calibration means that the measurement of the value of properties is related to measurements on primary standards usually provided by the primary national laboratory. The relationship is called traceability. The purpose of calibration and traceability is to ensure that all measurements are made with the same sized units of measurement to the appropriate level of uncertainty so that the results are reliably comparable from time to time and place to place.

Traceability is the ability to relate individual measurement results through an unbroken chain of comparisons leading to one or more of the following sources: national primary standards, intrinsic standards, commercial standards, ratios, and comparison to a widely used standard which is clearly specified and mutually agreeable to all parties concerned.

The concept of calibration may not be directly relevant for software. An international report of the open systems community, *ISO/IEC TR13233: 1995 Information technology—Interpretation of accreditation requirements in ISO/IEC Guide 25—Accreditation of Information Technology and Telecommunications testing laboratories for software and protocol testing services* [19], substituted the terms “validation and traceability” for the terms “measurement traceability and calibration.” The report concluded that validation is to software and protocol test tools as calibration is to measurement equipment.

NISTIR 6025 identified measurement and test areas that needed additional research and development to advance the state of IT metrology. Some of these areas are being explored, while others have not been studied or developed extensively.

*Level of confidence in test results*. Currently the quality of an information technology product or component is assured without rigorous metrics for confidence in test results. Commercial producers of software use a combination of factors such as code analysis, beta testing and defect analysis to determine that a product is “good enough” to release. Various analyses of code can be a step toward a more rigorous definition of certainty of a product’s quality, but more needs to be done to define the mathematical foundations and methods for assessing the uncertainty in quality determinations. IT metrology needs a set of concepts equivalent to calibration, traceability, and uncertainty, which are important in physical metrology. If uncertainty could be calculated by statistical methods as for physical test results, the level of confidence in software could be calculated.

*Interoperability testing*. Work is needed to investigate the implications of combinations of interoperability testing. For example, if implementation A and implementation B interwork and if implementation B and implementation C interwork, what are the prospects of implementations A and C interworking?

*Automatic generation of test code*. Developing test code for IT conformance testing can be more time consuming and more expensive than developing the standard or a product which implements the standard. Efforts are needed to specify more formally the standard or specification and to generate test code from this formalization.

*IT dimensioning or description system(s)*. NISTIR 6025 discussed general concepts of fundamental and derived units for IT metrology. These concepts could be expanded, and a general vocabulary could be developed to describe the components, which comprise information systems. This would entail developing a rich, standardized terminology to capture the functionality and capabilities of a software component, in addition to the interface specifications. The definition of these formal specifications in a standardized, rigorous way would enable designers and systems integrators to select software components with confidence regarding the component’s capabilities and how it will integrate into the system being built. Furthermore, automated composition of systems based on specifications will be possible once these types of definitions exist and are widely deployed in a certifiable way.

*Software metrics*. Software metrics are needed to more rigorously measure and test increasingly complex software as it is being developed.

*Algorithm testing*. As researchers develop new algorithms, some means of measuring the performance of these algorithms for comparison purposes is needed. The requirement is for measures of performance such as benchmarking programs, which are used to measure specific aspects of a computer’s capabilities. There are challenges: determination of a theoretical foundation for measuring the performance of algorithms, and means of ensuring that implementation-dependent performance results are meaningful.

NIST has been performing numerous activities to advance IT measurement and testing and to assist industry in the development and application of measurements and tests for IT. Following are descriptions and summaries of a few selected NIST projects in the areas of conformance testing, interoperability testing, performance testing, and reference data. These projects describe present needs in IT measurement and testing and NIST’s capabilities to address such needs.

### 3.1 Conformance Testing

The concept of *conformance testing* has been applied to IT for about thirty years, a relatively long period of time for IT. From *ISO/IEC Guide 2: 1996, Standardization and related activities—General vocabulary*, a definition can be derived as:

conformance testing

Systematic examination of the extent to which a product, process or service fulfills specified requirements by means of testing.

Conformance testing methodology is well developed and widely used. Testing methodologies have been developed for operating system interfaces, computer graphics, document interchange formats, computer networks, and programming language processors.

IT standards are almost always developed and specified in a natural language, English, which is inherently ambiguous. Sometimes the specifications are originally developed or translated into a less ambiguous language called a formal description technique (FDT). Because of the complexity and ambiguity, most testing methodology documents require the development of a set of test case scenarios (e.g., abstract test suites, test assertions, test cases) which must be tested.

The standards developing activity usually develops the standard, the FDT specification, the testing methodology, and the test case scenarios. Executable test code which tests the test case scenarios is developed by one or more organizations, often resulting in more than one conformance testing product being available. However, if a rigorous testing methodology document has been adhered to, it should be possible to establish whether each product for testing conformance is a quality product and an equivalent product. In some cases, an executable test code and the particular hardware/software platform it runs on become accepted as a reference implementation for conformance testing. Occasionally, a widely successful commercial IT product becomes both the de facto standard and the reference implementation against which other commercial products are measured.

NIST has been a leader in developing conformance testing methods and products for standards or specifications to promote the development of testable standards and specifications, the interoperability of products, and the development of conforming products. Current projects focus on testing and measurement to expedite the research, development, standardization, and commercialization of new technologies. The emphasis is placed on developing tests early in the technology cycle to help IT developers improve the design of their products.

#### 3.1.1 Standard for the Exchange of Product Model Data

**Fig. 2:**
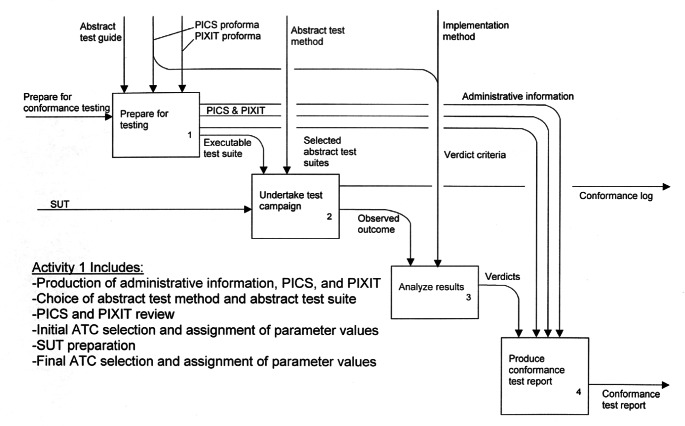
Conformance assessment process.

NIST/MEL has developed web-accessible conformance testing capabilities for use by software vendors seeking to implement ISO 10303, *Standard for the Exchange of Product Model Data*. ISO 10303 is the international standard for the exchange of product model data. This standard, commonly known as “STEP,” was developed to facilitate electronic communication among the engineering and manufacturing software systems used throughout the aerospace, automotive, and electronics supply chains [20]. NIST has played a significant role in industry’s development and adoption of Standard for the Exchange of Product Model Data (STEP) [21]. STEP is not a single standard but rather a complex suite of standards aimed at different data exchange scenarios. The architecture of STEP includes a language for information modeling, methods for mapping information representations to exchange structures, integrated resources with which to construct application-specific specifications, and the actual specifications that software vendors implement—known as Application Protocols (APs). STEP developers recognized the need to provide conformance specifications in conjunction with the APs early on. Conformance classes are specified as part of each AP, and Abstract Test Suites (ATSs) are called for as well. The conformance testing process as envisioned in the STEP standards is illustrated in Fig. 2.

Hence the standards community provided the basic notion with which to test the conformance of software implementing STEP. However, as a voluntary standards organization, the technical committees could not provide the remainder of the infrastructure necessary to conduct conformance testing. Specifically there was the need for unique software tools to facilitate STEP conformance testing, a laboratory to conduct the tests, and an organization to ratify the results of the tests. In fact, NIST, in conjunction with the Center for Electronic Commerce at the Environmental Research Institute of Michigan (CEC ERIM), had already developed many of the specialized tools necessary to conduct conformance testing through its earlier work in STEP interoperability testing. Industrial representatives from Boeing, General Motors, and others provided the motivation to initiate a conformance testing service through their participation in the U.S. Product Data Association (USPRO). Concurrent with the establishment of the STEP conformance testing service in USPRO, the French Operational Group for the Standard for Exchange and Transfer (GOSET) was also in the process of establishing a conformance testing service.

The USPRO STEP Certification service was formally announced in June 1998. Fundamental to the establishment of the service was the formation of a certification control board. The board was made up of representatives from the STEP user community (e.g., manufacturers such as Boeing and General Motors), vendors of STEP products (e.g., Parametric Technology Corp. and Unigraphics Solutions) and the standards community (i.e., the ISO subcommittee responsible for STEP, NIST). The role of the board was to set policy with regard to testing requirements, procedures, and to provide a forum for resolution of certification issues. NIST provided guidance in the establishment of the board and acted as liaison to the American National Standards Institute (ANSI) and the National Voluntary Laboratory Accreditation Program for accreditation questions.

**Fig. 3:**
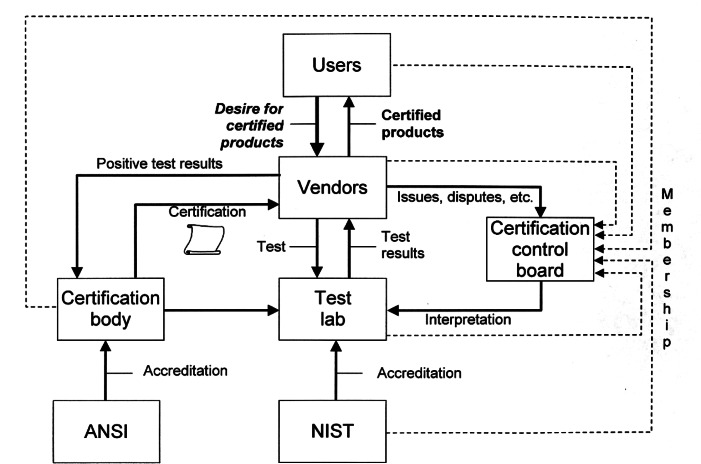
ISO 10303 certification process.

**Fig. 4:**
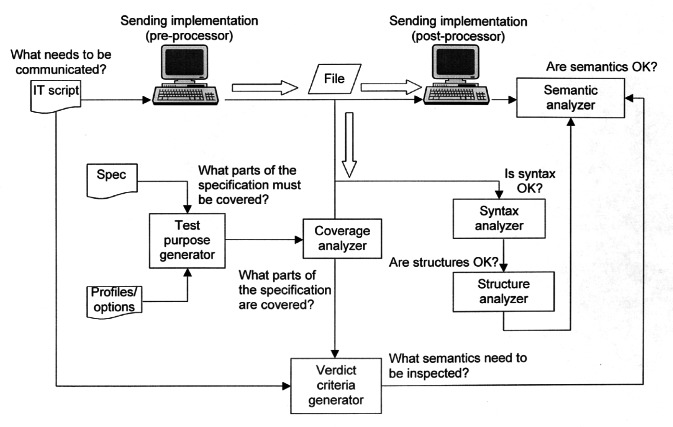
STEP conformance testing tools.

The certification process employed (shown in Fig. 3) is innovative in the sense that it is conducted via the Internet. Rather than having representatives come to a vendor’s physical facility to conduct tests, vendors download test cases from the test laboratory’s website, create the specified product representations in their systems, and upload their results to the test laboratory’s website where they are analyzed by the test laboratory personnel. These transactions are conducted in a secure environment and privacy is assured. The internet-enabled testing process also allows vendors to pre-test and debug their products in advance of the formal test. At the testing laboratory, CEC ERIM, a suite of unique software tools, is employed both to generate test data according to the conformance classes and test suites specified in the STEP standard as well as to analyze the results vendors submit. The tools and processes utilized at the testing laboratory are illustrated in Fig. 4.

The first USPRO certification was awarded in April 1999 to Dassault Systemes for their Computer Assisted Three-dimensional Interactive Application (CATIA) computer-aided design product.^1^ Since that time three other vendors’ products have been certified and there are additional certifications in progress. At the same time NIST has been working with GOSET to harmonize U.S. certification requirements and procedures with those offered in France. The expected result is that there will be mutual recognition of STEP certifications internationally.

#### 3.1.2 Cryptographic and Other Security Standards

Security standards have special requirements for testing to assure that implementations do not contain errors or flaws that could be used to attack systems. NIST/ITL has developed conformance tests for cryptographic standards that have been issued as Federal Information Processing Standards (FIPS). *The Data Encryption Standard* (DES) [22] was the first cryptographic standard for which ITL developed conformance tests. A testbed was designed to validate hardware implementations of the DES. An implementation was verified by ITL if it correctly performed a set of 291 test cases that had been defined to exercise every basic element of the DES.

Currently, ITL coordinates validation programs for the Federal Information Processing Standards (FIPS) that specify cryptographic functions. These standards include the *Data Encryption Standard* (DES), *Digital Signature Standard* (DSS) [23], *Secure Hash Standard* (SHS) [24], and *Security Requirements for Cryptographic Modules* [25]. The latter standard specifies the overall requirements for the modules that implement cryptographic algorithms and methods, and is the framework for all of the cryptographic standards. Before this standard was in place, security implementations often failed despite the use of strong encryption methods. NIST realized that standards for encryption algorithms were not enough, and that there was a need for a method to specify the characteristics of the cryptographic module. Cryptographic modules can be standalone equipment, add-on encryption boards embedded into a computer system, or software applications such as digital signature operations. When cryptographic logic is implemented in software, the processor that executes the software is also part of the cryptographic module.

NIST/ITL established the Cryptographic Module Validation Program (CMVP) to manage the validation testing of cryptographic modules and algorithms that are specified by FIPS. The CMVP has been carried out in cooperation with the Communications Security Establishment (CSE) of the Government of Canada since July 1995. The tests are conducted by third-party laboratories, which have been accredited as Cryptographic Module Testing (CMT) laboratories by the National Voluntary Laboratory Accreditation Program (NVLAP). Information on tested products is available to all. Products validated as conforming to standards are placed on a Validated Products List, and are available for use by the Federal agencies of the United States and Canada for the protection of sensitive information. The CMVP promotes the use of validated products and provides Federal agencies and the private sector with a security metric to use in procuring equipment containing cryptographic modules. Users selecting equipment on the Validated Products List can have confidence that the products meet their requirements for security. The program validates a wide variety of modules including Public/Private Key encryption products, secure radios, and tokens.

NIST and the National Security Agency (NSA) initiated a major effort to promote an internationally accepted testing program for security products through the National Information Assurance Partnership (NIAP). This joint activity is directed toward meeting the needs of information technology producers and users for tested security products. The products are tested against the *Common Criteria (CC) for Information Technology (IT) Security Evaluation*, an international standard (ISO/IEC 15408) [26] for specifying and evaluating the security features of computer products and systems. The Common Criteria (CC) can be applied to a wide range of technologies such as operating systems, database management systems, Public Key Infrastructure (PKI), firewalls, smart-cards, telecommunications switches, network devices, middleware, and applications. NIST, NSA, and the security organizations of Canada, France, Germany, the Netherlands, and the United Kingdom contributed to the development of the CC to replace the many different security evaluation methods that had been used in North America and Europe.

NIAP establishes objective test methods and testing procedures for the cost-effective evaluation of security products. Security evaluations are formalized testing and analytic processes that use the CC to determine whether IT products have been developed correctly according to their specifications and whether they are effective in countering stated security problems. The testing is done by accredited, independent testing laboratories. The goal is to enable product developers to get their products tested easily, and for users to have access to information about tested products. The evaluations help users to determine whether IT products and systems meet their security requirements.

#### 3.1.3 Extensible Markup Language (XML)

NIST/ITL is developing conformance tests for Extensible Markup Language/Document Object Model (XML/DOM) [27]. The success of the World Wide Web is due in a large part to the success of the Hypertext Markup Language (HTML), and its universal methods for displaying data. XML affords a standards-based approach to defining, interacting with, and exchanging data for a variety of domain-specific applications. DOM defines ECMAScript and Java1™ bindings for interacting with both XML and HTML data, permitting dynamic creation and manipulation of web pages defined using these metalanguages.

Although the XML specification [28] was released in 1999 by the World Wide Web Consortium (W3C), there are already millions of XML pages appearing on the Web. Virtually all application domains are looking to use XML to exchange structured information. In addition, XML processors are beginning to appear in beta versions of popular web browsers and associated applications. As such, conformance of these XML processors to the W3C Recommendation will permit robust, interoperable solutions. NIST/ITL, in conjunction with industry partners, has developed a robust test suite that will be used as a metrology tool for testing XML processors.

### 3.2 Interoperability Testing

A generic definition for *interoperability testing* is:

*interoperability testing* (NISTIR 6025)

The testing of one implementation (product, system) with another to establish that they can work together properly.

Interoperability testing methodologies are not as well established as conformance testing methodologies. Interoperability testing usually takes one of three approaches to ascertaining the interoperability of implementations (i.e., commercial products). The first is to test all pairs of products. Typically an IT market can be very competitive with many products and it can quickly become too time consuming and expensive to test all of the combinations. This leads to the second approach of testing only part of the combinations and assuming the untested combinations will also work together. The third approach is to establish a reference implementation and test all products against the reference implementation.

NIST develops methodologies and tools to facilitate interoperability testing and pilot deployment of emerging technologies. Interoperability testing is typically focused on the early stages of product research and development. NIST helps industry conduct interoperability testing by providing reference implementations and testing tools; and by often facilitating multi-vendor interoperability testing events and testbeds.

#### 3.2.1 IPsec Web-based Interoperability Tester

NIST/ITL developed the Internet Protocol security Web-based Interoperability Tester (IPsec-WIT) in response to the need of the Internet Engineering Task Force (IETF) for an interoperability test system for Internet security protocols. The IPsec-WIT supports the current Internet protocol suite as well as the future addition of new functionality to the Internet Protocol suite (IPS) as the use of the Internet continues to increase. IPsec was developed around the Cerberus prototype. Cerberus is a reference implementation of security protocols developed by the Internet Engineering Task Force (IETF), and used by many companies as a basis for product development and testing. Implemented as a kernel module for the Linux operating system, the Cerberus prototype supports IPsec authentication and encryption services in both tunnel (i.e., firewall) and transport (i.e., host) mode.

IPsec-WIT provides an innovative method [29] that allows implementers and system integrators to remotely control and execute a series of interoperability tests anytime and anywhere without requiring any distribution of test system software, relocation of the systems under test, or coordination of access to the reference implementation or tester. Users include security researchers, product implementers and highly technical users such as network engineers and administrators. This user community is primarily interested in easily available and flexible test systems that provide detailed technical diagnostics as systems and new applications are in the development phase.

**Fig. 5:**
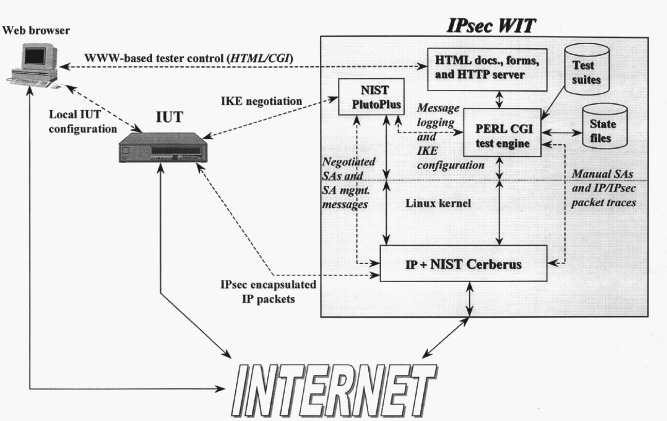
Ipsec-WIT architecture.

IPsec-WIT is a novel use of World Wide Web (WWW) technology for the purposes of protocol interoperability testing (see Fig. 5). The Web, or WWW, enables universal browsing among data repositories via the Internet. The Web has been used as a testing tool to test other WWW related technology [30], and as a simple configuration and control interface for remote protocol implementations. Using a common web browser, an IPsec-WIT user can easily navigate through directories of pre-defined test cases, configure the test system, execute tests, and view detailed test results.

The test system is based upon NIST developed, publicly available reference implementations of IPsec (*Cerberus*) and Internet Key Exchange (IKE) (*PlutoPlus*). Cerberus [31] is a Linux kernel reference implementation of the IPsec [32] protocol, including both the Encapsulating Security Payload (ESP) [33] and Authentication Header (AH) [34]. ESP and AH are encapsulation protocols that provide cryptographic confidentiality (ESP), and authentication (AH), to individual IP packets. PlutoPlus [35] is a Linux reference implementation of IKE [36], a dynamic key management protocol used to negotiate security associations (SAs) and keying material between two systems. Fundamentally, the IPsec-WIT system is a wrapper and front end that drives the Cerberus and PlutoPlus reference implementations and analyzes their output.

Cerberus and PlutoPlus reference implementations were designed to be completely operational stand-alone prototypes. They were extended with additional control and diagnostic capabilities specifically for use in IPsec. PlutoPlus is a complex, stateful key management daemon. This complexity allows for interoperability problems to occur at many different places in the protocol. Testing diagnostic statements were added extensively to the PlutoPlus implementation. To allow the test system to be used simultaneously by multiple users, PlutoPlus had to be modified to operate in a single negotiation mode in addition to its normal daemon mode. In single negotiation mode, the PlutoPlus process exits after each IKE negotiation (i.e., a single test). This allows the IPsec-WIT test system to independently control IKE behavior and correlate outputs for each user of the system.

The main engine of the IPsec-WIT system is a Perl cgi-bin script that ties the user interface HTML forms, directories of test cases, and prototype reference implementations into a working interoperability test system. Interoperability testing scenarios usually involve an iterative series of quick tests and long debugging periods. To allow users to retain state regarding test configurations and progression through test directories between debugging sessions, simple state files were developed to contain control parameters (e.g., tester configuration variables, Implementation Under Test (IUT) addresses, current test case, etc.). These files are indexed by a user provided unique user-id / password.

Test cases were customized with the user specific control information in the state files. The test cases are the primary component of this system. To deal with protocols with many optional features, it was necessary to organize the test cases into context relevant categories. For IPsec, the majority of interoperability problems are the result of non-interoperable or incorrect application of the cryptographic algorithms. Because of this, the IPsec test cases were organized into test directories based upon cryptographic algorithm (e.g., DES, 3DES, etc.). Organized in this way, users can easily test variations of a particular algorithm before moving on to the next algorithm.

Interoperability issues with IKE were explored. In any IKE negotiation there are several message exchanges involving multiple cryptographic algorithms. A failure at any point in the exchange will cause interoperability problems. IKE test directories were developed based on the different cryptographic algorithms, and the ability to make additional configuration changes to a given test was provided. This allows the users the flexibility to test for a given negotiated algorithm and then make subtle changes to the test configuration to test for more IKE specific problems.

The test cases serve as both documentation and executable code. The test cases were written in HTML to easily convey to the user the test purpose, how to configure the IUT for the test, and exactly how the test will be executed. The test cases were enriched with special tagged variables that the Perl script replaces with user specific control information, creating a test case tailored for a particular user.

The test system evolved from an IPsec test system with a handful of test cases to a robust test system that includes hundreds of IPsec and IKE test cases. This evolution resulted from the continued development of the NIST prototype implementations, feedback from IPsec-WIT users, changes and clarifications in the IPsec and IKE specifications, observations on how IPsec-WIT was being used, and new ideas about the test system.

Multi-vendor interoperability has been the corner stone for the explosive growth of the Internet. The increasing complexity of new protocols, such as that found in Internet security protocols, makes developing interoperable products very difficult. IPsec-WIT helps implementers and standards organizations work toward the development of interoperable products that are effective and cost-effective. More than 350 implementers and users have used IPsec-WIT to improve their formal testing procedures. The use of this system paves the way toward the development of testing tools for other protocols as well.

#### 3.2.2 STEP Interoperability Testing

**Fig. 6:**
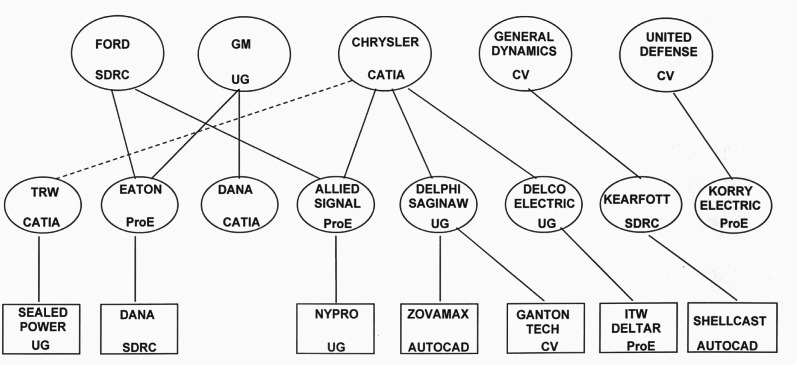
STEP interoperability testing.

From 1995 through 1997 NIST participated in the AutoSTEP project with the Automotive Industry Action Group (AIAG). The purpose of the AutoSTEP project was to introduce STEP into automotive industry usage, ensure that STEP met the needs of the industry, and to determine the benefits the industry could derive from using the standard [37]. The scope of the project was the exchange of product data for mechanical design using the STEP Application Protocol for configuration controlled 3D designs of mechanical parts and assemblies [38] for the purpose of packaging analysis. Participants in the project included original-equipment manufacturers (OEMS), first and second tier suppliers, software vendors and consultants. Figure 6 illustrates the tier relationships among the manufacturers involved along with the computer-aided design (CAD) systems utilized by each participant. The fundamental issue that had to be addressed by the AutoSTEP project was whether or not STEP would be an improvement over the data exchange methods then employed by the participants. That is, would it result in a higher rate of successful file transfers? Would it be able to transfer data that other methods could not? If STEP could be shown to reduce the costs of data exchange, save time, or overcome technical barriers in supply chain relationships, there would then be a business case for STEP adoption in this industry segment.

**Fig. 7:**
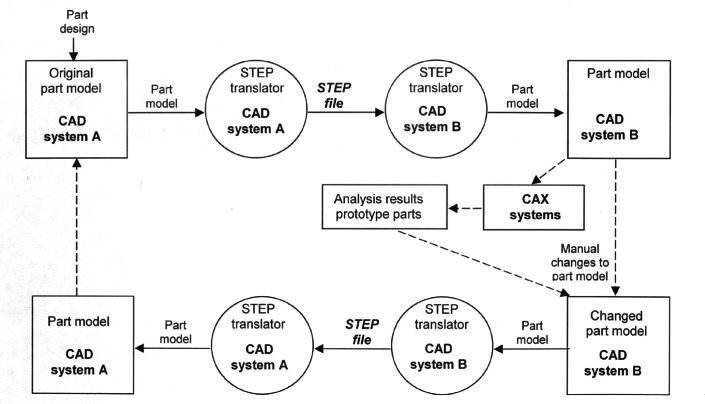
Model exchange scenario used in the AutoSTEP project.

To assess the efficacy of STEP for the project, participants defined meaningful metrics for OEM-supplier data exchange scenarios (see Fig. 7). These metrics included simple metrics that could be computed directly from the model exchanged as well as process metrics that are determined by comparing simple metrics at different stages in the exchange process. Some of the simple metrics could be determined using functionality available in the CAD systems being employed. Other required unique software tools developed by CEC ERIM in conjunction with NIST specifically targeted testing of STEP implementations. Among the metrics utilized in the project were:

- counts of different kinds of entities transferred in each exchange;
- properties of the model transferred such as its center of gravity, volume, and mass;
- counts of violations of constraints specified in the STEP standard;
- file size;
- translation time;
- the time required to make a model imported via STEP into a valid model in the receiving CAD system;
- the ratio of the time required to make an imported model valid versus the time required to recreate the original model in the receiving CAD system.

Over the course of the project the rate of successful file exchanges using STEP improved dramatically, exceeding 80 %. As the success rate began to plateau, factors not directly related to STEP began to emerge as impediments to successful exchange. Chief among these were the issues of CAD system accuracy and bad models. The accuracy issue was due to the mismatch in numerical accuracy maintained within different vendors’ products. Hence a model that is inherently accurate in one system (e.g., a model that forms a closed volume) because of that vendor’s maintenance of, say, 10^−6^ numerical accuracy may not form a closed volume in a system that only maintains accuracy of 10^−3^. Related to this issue was that of bad models. In one system a model may be deemed valid despite seemingly minor representational idiosyncrasies. Those same subtleties could cause the receiving system to declare the model as invalid. Errors of this type were initially attributed to problems with STEP translation but further investigation revealed their true pathologies. In the end, the AutoSTEP achieved its goals of demonstrating the efficacy of STEP for OEM-supplier data exchanges and this has resulted in use of the standard for actual commercial manufacturing.

### 3.3 Performance Testing

The *IEEE Standard Glossary of Software Engineering Terminology*, IEEE 610.12-1990 [39], defines *performance testing* as:

performance testing

Testing conducted to evaluate the compliance of a system or component with specified performance requirements.

Tools and techniques have been developed by NIST to assist in the performance measurement and characterization of networking technologies, middleware, distributed systems, and hardware components. The focus is on the research and development of the measurement methodologies and the characterization of generic technologies rather than the benchmarking of specific products or implementations.

#### 3.3.1 Manufacturer’s CORBA Interface Testing Toolkit

NIST/MEL is developing a publicly available software toolkit that supports several kinds of testing (integration, conformance, performance) of manufacturing system software components whose interfaces use the Object Management Group’s specification, the Common Object Request Broker Architecture (CORBA).

As part of NIST’s laboratory efforts in conjunction with the Advanced Technology Program’s Advanced Process Control Framework Initiative [40] and the National Advanced Manufacturing Testbed Framework project [41] a software toolkit was developed to address testing issues associated with distributed object architectures. Specifically these projects were employing the Common Object Request Broker Architecture (CORBA) [42] as the infrastructure upon which software components for manufacturing control were being built. While the CORBA infrastructure provides many advantages for implementation of distributed systems, testing the systems produced is difficult. Because components in such systems have complex interdependencies, testing the components in isolation is impossible. Hence NIST developed the Manufacturer’s CORBA Interface Testing Toolkit (MCITT, pronounced “M-kit”) [43]. MCITT minimizes the effort needed to produce simple emulations of servers, or dummy components that can be used to replace actual servers in a testing scenario. The toolkit is available from NIST [44].

Two complementary mechanisms are provided in the toolkit for defining the behaviors of dummy components. Each was developed specifically for use with MCITT and allow the toolkit user to specify CORBA server, client, or overall system interactions. The Interface Testing Language is a procedural language for specifying and testing the behavior of CORBA clients and servers. Interface Testing Language allows for run-time assertions, script-like behavior specifications, conformance testing commands, timed loops, as well as simple commands for creating and binding to CORBA objects. The second mechanism provided is the Component Interaction Specification (CIS). A CIS is a textual specification of an interaction scenario for an entire distributed system. Unlike an Interface Testing Language file, a CIS can describe nearly all of the interactions between all of the components in the system. One caveat regarding CIS capabilities is that it does not support the generation of client requests, though those can be added manually using Interface Testing Language. Together these two mechanisms provide a powerful arsenal with which to conduct a variety of tests in distributed systems based on CORBA. Unit testing, integration testing, system testing, performance testing, conformance testing, and stress testing are all possible using MCITT.

#### 3.3.2 NIST Network Emulation Tool

The NIST Network Emulation Tool (NIST Net) is a general-purpose tool for emulating the performance dynamics in IP networks. NIST Net was designed to allow controlled, reproducible experiments with network performance sensitive applications and protocols in a simple laboratory setting. By operating at the Internet Protocol (IP) level, NIST Net can emulate the critical end-to-end performance characteristics imposed by various wide area network situations (e.g., congestion loss) or by various underlying subnetwork technologies (e.g., asymmetric bandwidth situations involving (generic) Digital Subscriber Line (xDSL) and cable modems).

NIST Net is implemented as a kernel module extension to the Linux operating system and an X Window System-based user interface application. In use, the tool allows an inexpensive PC-based router to emulate numerous complex performance scenarios, including: tunable packet delay distributions; congestion and background loss; bandwidth limitation; and packet reordering/duplication. The X interface allows the user to select and monitor specific traffic streams passing through the router and to apply selected performance “effects” to the IP packets of the stream. In addition to the interactive interface, NIST Net can be driven by traces produced from measurements of actual network conditions. NIST Net also provides support for user defined packet handlers to be added to the system. Examples of the use of such packet handlers include: time stamping / data collection, interception and diversion of selected flows, generation of protocol responses from emulated clients.

#### 3.3.3 Exchange of Fingerprint Images

Since the late 1960s, the Federal Bureau of Investigation (FBI) and NBS/NIST have participated in a collaborative effort to automate the Federal Bureau of Investigation’s (FBI) fingerprint identification processing and to develop standards for the exchange of fingerprint information [45]. Until 1990 the standard fingerprint card was the primary source and repository for all fingerprint images. To automate the process for transmission, search, and identification of digital fingerprint images, the FBI decided to place their existing and projected master file of 400 million plus fingerprint images online. The typical size of a file needed to contain a single fingerprint image was estimated to be approximately 600 000 bytes. To make the use of these fingerprint images practical, each image file had to be considerably reduced in size to minimize storage space and transmission time resource demands. The Wavelet Scalar Quantization (WSQ) algorithm was developed as the compression technique to be used. This lossy compression approach easily produces a 15:1 size reduction with minimal loss in image fidelity.

**Fig. 8:**
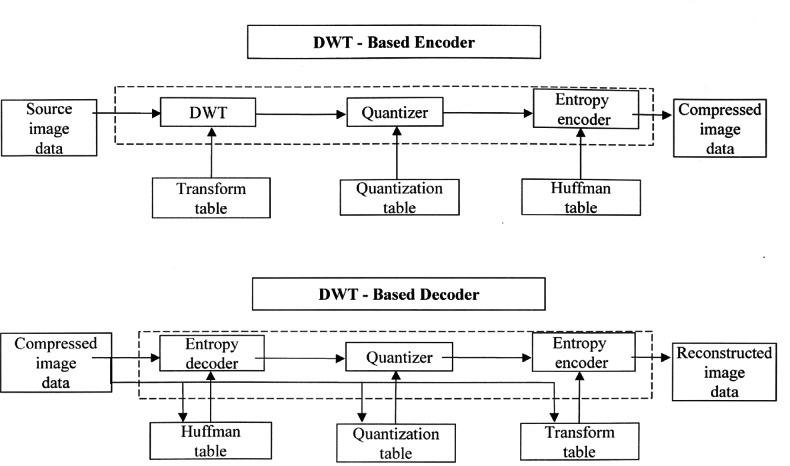
WSQ encoder/decoder overview.

The WSQ specification [46] outlines a comprehensive and flexible framework for compressing gray-scale fingerprint images using the wavelet scalar quantization technique. Figure 8 is an overview of the operation of the WSQ encoder and decoder. The encoder uses the source image as input. It then performs a decomposition of the fingerprint image into a number of subbands, each of which represents information in a particular frequency band. This decomposition is accomplished by a discrete wavelet transformation of the fingerprint image. Each of the subbands is then quantized using values from a quantization table where the “lossy” attribute of the algorithm and resulting data compression is introduced. These quantized coefficients are then passed to a Huffman encoding procedure that produces the final compressed image file. The decoder portion of the algorithm essentially operates in reverse. A compressed image is the input to the decoder. The Huffman, quantization, and transform tables are extracted from the compressed file and used in the entropy decoder, dequantizer, and transform steps to produce a reconstructed image.

To ensure adherence to the specification, NIST/ITL established a compliance testing program for vendors and researchers to follow. An encoder implementation must produce a syntactically and numerically correct compressed file in accordance with the WSQ algorithm. The compressed file must be capable of being properly decoded without any errors by an independent decoder implementation. A decoder implementation must be capable of correctly reconstructing an image file from a syntactically correct WSQ compressed file. When compared to the original image file, the reconstructed image file should be numerically accurate to within specified tolerances.

Validation for a specific WSQ implementation is based on compressing a reference set of test images in accordance with the WSQ specification. The tests specified for encoders and decoders are not exhaustive tests of interoperability, numerical precision, or image fidelity, but tests of correctness of the images tested. It is not possible to test all images of all arbitrary sizes, using all the features contained in the specification. The ITL approach tests only part of all the possible combinations that can be used and assumes that untested combinations will also function properly.

Compliance with the WSQ specification is determined by comparing the output from the candidate implementation with the output from a double precision reference implementation developed by NIST/ITL. Products are evaluated using this same reference implementation. The criteria for product validation use the test results from candidate WSQ implementations to determine if acceptable tolerance limits have been met. Tolerance limits were derived by comparing the results from the ITL reference implementation with results from an implementation developed by the Los Alamos National Laboratory. The two implementations were developed independently but in the same time frame. Intermediate results on 1800 processed fingerprint images from Special Database 9 [47] were compared between the two implementations and the results were used to determine the acceptable tolerances.

**Fig. 9:**
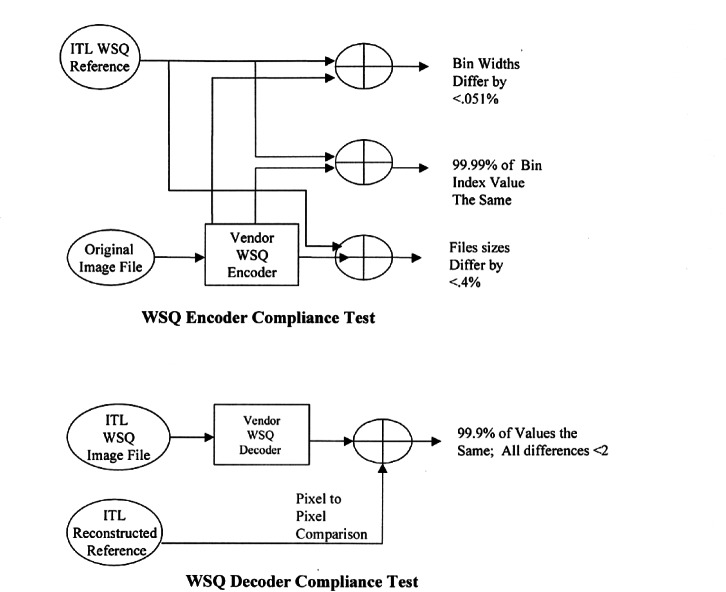
WSQ encoder and decoder compliance tests.

To have a WSQ encoder/decoder implementation validated, the product developer downloads the ITL reference test set from the Internet. This test set consists of 17 fingerprint images. Each image was scanned at a resolution of 19.69 pixels per millimeter (500 pixels per inch) and quantitized to 8 bits of gray-scale (256 levels). Dimensions of the image sizes ranged from 375 × 526 pixels to 832 × 768 pixels. In addition to the raw scanned image file, a WSQ-encoded image file, and a reconstructed image file is provided for each of the original fingerprint image files. The developer returns two files for each of the 17 image files. Output data sets generated by a candidate implementation must match the output data sets from the ITL reference implementation to within the stated accuracy requirements (see Fig. 9).

All federal, state, and local agencies doing business with the FBI are required to transmit and receive fingerprints as compressed fingerprint image files. The implementation validation program aims to ensure that interoperability, proper numerical precision, and image fidelity are consistent among different implementations. When the compressed image files created for exchanges are in accordance with the WSQ Gray-scale, fingerprint images can be exchanged between federal, state and local agencies. These organizations require that vendors comply with the Fingerprint Image Compression Specification and vendors obtain validation for their implementations of the WSQ algorithm. To date, over one hundred implementations have been submitted and tested for compliance to the WSQ algorithm. Companies who have participated in this program include Aware, Cogent, Harris, IBM, PRC, Printrak, and Sagem-Morpho. As a result of this program, testing for proper performance of fingerprint image systems has achieved reliable interoperability between the systems of FBI and of different agencies using dissimilar Automated Fingerprint Identification Systems (AFIS).

### 3.4 Reference Data

The use of *reference data* is very important in both physical and IT metrology. But the term has different definitions for reference data as applied to physical and IT metrology:

*reference data* (NISTIR 6025)

In physical metrology, reference data is quantitative information, related to a measurable physical or chemical property of a substance or system of substances of known composition and structure, which is critically evaluated as to its reliability.

In information technology, reference data is any data used as a standard of evaluation for various attributes of performance.

Reference data provide critical information for testing the performance of complex IT systems. For many kinds of IT systems, NIST has played an important role in collecting and disseminating unbiased data that has been used by and relied upon by developers to test the performance of their systems.

#### 3.4.1 Algorithm Testing and Evaluation Program for Coordinate Measuring Systems

NIST/MEL is identifying reference data to be used in the testing of coordinate measuring machine algorithms and to compare the performance of a coordinate measuring machine with the performance of other machines in a similar category. MEL has developed a software system, which can be used to assess the uncertainty in coordinate measuring machine algorithms.

Coordinate measuring systems (CMSs) are machines used by manufacturers to determine dimensional characteristics of mechanical parts by analyzing 3-dimensional point data acquired on part surfaces [48]. CMSs employ data analysis software to reduce measured point coordinates to curve and surface geometries. In turn, these geometries are compared to the tolerance limits for the part. It is the uncertainty of the geometries computed by the CMS data analysis software that determines the quality of the measurement. In the past decade mounting evidence showed that data analysis software had significantly contributed to measurement errors in CMSs. To help address the issue of data analysis software quality, NIST developed an Algorithm Testing System for CMS software and provides a Special Test Service through the NIST Calibration Program to assess the uncertainty of CMS software. The test service is known as the Algorithm Testing and Evaluation Program for Coordinate Measuring Systems (ATEP-CMS).

**Fig. 10:**
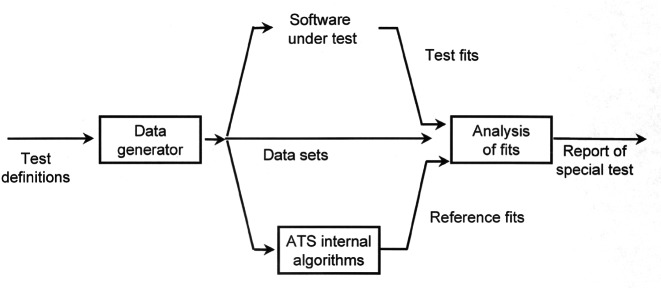
Architecture of the NIST algorithm testing system.

The architecture of the NIST Algorithm Testing System is illustrated in Fig. 10.

The data generator is driven by a test description containing a high level description of the tests to be performed. The generated data is then provided both to NIST’s highly accurate internal algorithms [49] and to the CMS software under test. NIST’s algorithms yield reference results to which the test fits returned by the software under test are compared. The analysis compares geometric differences among fits and detects code errors and systematic bias in the CMS software. Test descriptions can be tailored to estimate different aspects of CMS software errors.

The ATEP-CMS is the first calibration service ever to be offered by NIST for software. NIST’s experience with ATEP-CMS is assisting standards committee in the American Society of Mechanical Engineers (B89.4.10, Methods for Performance Evaluation of Coordinate Measuring System Software) and in the International Organization for Standardization (ISO) (TC213 WG10, Dimensional and geometrical product specifications and verification) on development of CMS test procedures and test data sets. In addition, NIST is currently working to make the ATEP-CMS a web-accessible calibration service.

#### 3.4.2 Speech Corpora

For more than a decade, NIST/ITL has been involved in developing reference data of standardized speech and natural language to help the research community, industry, and universities measure and improve speech recognition algorithms and to facilitate error analysis and diagnostics [50]. Initially, the collections of speech data were recorded on 9-track magnetic tapes, which often proved unreadable. Later experiments used a hybrid system involving Beta and Video Home System (VHS) format video tapes on which Pulse Code Modulation (PCM) files were recorded. This was replaced by Digital Audio Tape (DAT) and then by Compact Disk—Read Only Memory (CD-ROM).

The reference data that has been recorded and distributed on CD-ROMs contains speech databases, referred to as “corpora.” These corpora are collections of digitized waveforms, annotations, transcriptions, and related data, and include “found speech” from radio broadcasts as well as “read speech,” readings of prepared text. This work has been done in cooperation with the Defense Advanced Research Projects Agency (DARPA).

Many developers of speech recognition and speech understanding applications use the speech corpora to refine their systems. In cooperation with developers and research institutions, ITL has prepared development and evaluation test sets for speech technology applications. The technology developers apply the tests and then provide ITL with the results of the tests that they have implemented. ITL analyzes the data, and prepares uniformly-scored tabulated results, including the results of numerous paired-comparison statistical significance tests and other analyses. These results assist developers in improving the quality of their systems.

#### 3.4.3 Text Retrieval Conferences (TREC)

Text Retrieval Conferences (TREC), sponsored by NIST and the Defense Advanced Research Projects Agency (DARPA), were started in 1992 to support research within the information retrieval community [51]. The conferences provide the infrastructure necessary for large-scale evaluation of text retrieval methodologies. The TREC conferences encourage research in information retrieval based on large test collections, and help to increase communication among industry, academia, and government by creating an open forum for the exchange of research ideas. As a result, technology can be transferred more quickly from research labs into commercial products. The conferences have helped to demonstrate substantial improvements in retrieval methodologies on real-world problems and have increased the availability of appropriate evaluation techniques for use by industry and academia.

TREC is overseen by a program committee, which is composed of representatives from government, industry, and academia. NIST provides a test set of documents and questions. Participants run their own retrieval systems on the data, and return to NIST a list of the retrieved top-ranked documents. NIST pools the individual results, judges the retrieved documents for correctness, and evaluates the results. The TREC cycle ends with a workshop that is a forum for participants to share their experiences.

This evaluation effort has grown in both the number of participating groups and the number of tasks each year. The conference held in 1999 included 66 groups representing 16 countries. The TREC test collections and evaluation software are available to the retrieval research community at large, so organizations can evaluate their own retrieval systems at any time. TREC has successfully met its dual goals of improving the state-of-the-art in information retrieval and of facilitating technology transfer. Retrieval system effectiveness has approximately doubled in the 7 years since the first conference. TREC has also sponsored the first large-scale evaluations of the retrieval of non-English (Spanish and Chinese) documents, retrieval of recordings of speech, and retrieval across multiple languages. The TREC test collections are large enough so that they realistically model operational settings, and most of today’s commercial search engines include technology first developed in TREC.

## 4. Research and Development for IT Measurement and Testing at NIST

Work on advanced methodologies and techniques is directed toward improving the quality of testing and measurement in areas such as software testing and devising new approaches and tools to test technologies such as collaborative systems.

### 4.1 Advanced Encryption Standard

Strong, reliable cryptographic algorithms are essential for protecting sensitive data and electronic transactions in the 21st century. To replace the cryptographic algorithms that have been available to protect information throughout the 1980s and 1990s, NIST/ITL invited voluntary standards organizations, the cryptographic community, and industry to help define requirements for, to develop, and to evaluate candidates for the new cryptographic algorithm. This unique participatory public process started in 1997 is expected to result in an Advanced Encryption Standard (AES) that will be completed in 2001. The AES will specify an unclassified, publicly disclosed encryption algorithm capable of protecting sensitive government information well into the 21st century.

In 1998, 15 candidate algorithms were accepted for analysis for suitability for the AES. The assistance of the cryptographic research community was solicited in analyzing the candidates. The review of each algorithm included a methodical evaluation of the following characteristics: security (including any known attacks or weaknesses); efficiency (both speed and memory usage); flexibility (implementation on low- and high-end smart cards; support of additional key and block sizes, including whether the reference code actually supported the additional key sizes; suitability for use as a pseudo-random number generator, hashing algorithm, etc.; and whether or not encryption and decryption were the same procedure); algorithm simplicity; and other issues raised by the public discussion.

Security was the most important factor in the evaluation, and it encompassed features such as: resistance of the algorithm to cryptanalysis, soundness of its mathematical basis, randomness of the algorithm output, and relative security as compared to other candidates.

Cost was the second important area of evaluation, encompassing licensing requirements, computational efficiency (speed) on various platforms, and memory requirements. The speed of the algorithms on a wide variety of platforms was considered. The focus was primarily on the speed associated with 128 bit keys. Additionally, memory requirements and constraints for software implementations of the candidates were considered.

The third area of evaluation included characteristics such as flexibility, hardware and software suitability, and algorithm simplicity. Flexibility included the ability of an algorithm to handle key and block sizes beyond the minimum that must be supported; to be implemented securely and efficiently in many different types of environments; and to be implemented as a stream cipher, hashing algorithm, and to provide additional cryptographic services. It must be feasible to implement an algorithm in both hardware and software, and efficient firmware implementations are advantageous. The relative simplicity of an algorithm’s design was also an evaluation factor.

Tests were devised to measure the characteristics and to analyze the algorithms in these areas of evaluation. In addition to its measurements and evaluations, NIST considered all comments, papers, studies, reports and proposed modifications. Each candidate was reviewed relative to the announced evaluation criteria and other pertinent criteria suggested by the public. The results of the research were reviewed with the cryptographic community and five algorithms were selected for further study. After the five candidate algorithms were selected, the results of all of the analyses were made public, and further tests were devised to select the algorithm or algorithms to be considered for the AES. On October 2, 2000, NIST proposed the Rijndael data encryption algorithm as the AES. NIST will prepare the draft AES FIPS for public review and comment. After the close of the public comment period, the standard will be revised by NIST, as appropriate, in response to those comments. A review, approval, and promulgation process will then follow. If all steps of the AES development process proceed as planned, it is anticipated that the standard will be completed by the summer of 2001 [52].

### 4.2 Random Number Generation

The need for random and pseudorandom numbers arises in many cryptographic applications. For example, common cryptosystems employ keys that must be generated in a random fashion. Many cryptographic protocols also require random or pseudorandom inputs at various points, e.g., for auxiliary quantities used in generating digital signatures, or for generating challenges in authentication protocols. A study of random number generation was undertaken to investigate whether statistical testing could be applied to cryptographic testing. The study was published as NIST Special Publication 800-22 *A Statistical Test Suite for Random and Pseudorandom Number Generators for Cryptographic Applications* [53], and discusses some aspects of selecting and testing random and pseudorandom number generators.

Random number generators used in cryptographic applications may need to meet stronger requirements than for use in other applications. In particular, their outputs may need to be unpredictable in the absence of knowledge of the inputs. The criteria for characterizing and selecting appropriate generators were examined as well as the possible use of statistical testing instead of cryptanalysis. Although statistical tests were found to be useful as a first step in determining whether or not a generator would be suitable for a particular cryptographic application, no set of statistical tests could absolutely certify a generator as appropriate for use in a particular application. Therefore, it was concluded that statistical testing is not an adequate substitute for cryptanalysis.

### 4.3 Software Testing Methods

NIST/ITL is investigating methods to produce software tests, including conformance tests, which are derived from formal specifications. Specification based testing methods are not well developed, but they promise significantly more economical means for testing software than currently available, and reduced time-to-market for companies producing software products. Most software testing has focused on structural testing, that is, testing based on execution paths within the code that implements a specified function. There are so many possible combinations that testing all of them is not feasible. Further, structural testing is not possible with many systems, as there is no access to source code. Deriving tests from specifications is particularly useful for bodies developing software standards, e.g., the World Wide Web Consortium (W3C), the Object Management Group (OMG), the Institute of Electrical and Electronics Engineers (IEEE). The goal of these groups is specification development, while the goal of the participating companies is software implementation. Companies implementing software to standard specifications will be able to improve software quality by automatically generating software tests and demonstrating conformance to them. As a result, software products with fewer failures and closer adherence to standards can come to the market earlier and at less cost.

Another approach to improving software system quality is through the collection and analysis of error, fault, and failure data. The error, fault, and failure data are being used to develop profiles for industry use and for improving statistical analysis methods. A handbook of faults with detection and prevention methods is being developed. Software tools are being developed to enable software developers to collect and analyze data. These data collections help to fill a need for reference data on software failures to protect against the release of software systems with faults and to help assess software system quality.

Further work to improve the development and measurement of software testing is through the application of statistical methods to derive quantitative measures of software correctness or quality. Under investigation and development are new methods for software testing based on stochastic processes and statistical measures to provide qualitative measures for determining the probability that software correctly adheres to its specification. This includes both white-box testing and black-box testing to determine if a program conforms to its functional specifications. White-box testing assumes that program source code is available for inspection and metering. Black-box testing allows access to a program only through its defined interface. Functional specifications developed under conventional techniques usually specify formal syntax with semantic rules written in natural language. Functional specifications developed through formal methods require that both syntax and semantics be specified using mathematically rigorous techniques. The goal is to integrate statistical techniques into software testing to ensure software quality and to provide quantitative measures of stability, reliability, and conformance to specifications.

### 4.4 Testability of Interaction-driven Manufacturing Systems (TIMS)

NIST/MEL is characterizing the performance of software systems (e.g., CORBA) in the context of real-time manufacturing requirements. This project focuses on methods for testing multiple software components interacting with each other according to specified behaviors. This includes investigating mechanisms to build testability into software behavior specifications.

An interaction-driven manufacturing system is one composed of multiple software components in which the interactions between those components are automated. Automation is typically achieved through the definition of interfaces, both standard and proprietary, which allow the components to directly interact without the need for human intervention at every step along the way [54]. Such a system is typically implemented in a distributed fashion and its primary function is organized around the interactions of complex, loosely coupled components. The components may be large and complex systems themselves, they may be proprietary commercial off-the-shelf systems whose inner workings are not knowable, and they may purport to support standard interfaces. Since interaction-driven systems had begun to appear frequently in the manufacturing context, a project to respond to the growing need for relevant testing expertise was begun in 1998. The mission of the TIMS project is to close the gap between testing capabilities and the testability of interaction-driven manufacturing systems. The project is striving to enhance testing capabilities with improved test methods, by defining design-for-testability criteria for specification writers, and by applying tools and techniques to improve the quality of system-level specifications [55].

Thus far the project has investigated the characteristics of interaction-driven manufacturing systems, investigated mechanisms for specifying interactions [56], conducted experimental testing of systems, and identified testability issues as a result of those experiments. A significant amount of work has been spent developing integrated testing capabilities for software implementing the Object Management Group’s Product Data Management Enablers specification [57] or the related Product Data Management (PDM) Schema being developed for the international STEP standard [58] [59]. In essence these emerging standards are duals of each other and share a common semantic notion of PDM data. These standards would be implemented by PDM software vendors thereby making their PDM systems available as components of larger software systems. In developing test capabilities, the TIMS project uncovered numerous issues with respect to the testability of the specifications developed by the standards community [60], and this experience will help drive anticipated work on design for testability. In addition, the project has also conducted experiments in identifying faults that are introduced by imperfect integration of legacy components for control of a manufacturing shop floor and in testing of agent-based systems in a manufacturing scenario. Project results and the status of on-going efforts are kept up-to-date on the project’s web pages.

## 5. The Future of IT Metrology

At the start of the 20thcentury, the industrial age was well underway. Electricity and the electric light, the automobile, the telephone, the railroad and the telegraph lines connecting the Nation characterized the industrial infrastructure. The establishment of the National Bureau of Standards in 1901 was a consequence of the realization that scientific and industrial progress in the new century would require better measurements in the laboratory and the factory. Now, at the start of the 21st century, we are in the midst of an information age. Therefore, it certainly seems most appropriate to consider where IT will be in 10 years and how that might drive requirements for IT measurements by 2010.

### 5.1 The Forecasting Dilemma

However it is daunting to forecast 10 years ahead for IT because of the ongoing pace of innovation in IT and the rates of deployment of new IT. Software product lifecycles are now measured in web years (1 web year = 90 calendar days) so looking ahead 10 calendar years is actually looking ahead 40 web years, a tricky business at best. A decade ago, no one foresaw the explosion of the deployment of the Internet and the creation of the World Wide Web. Presently, traffic on the Internet doubles every 100 days. The next 10 years in IT are most likely to continue to be characterized by unforeseen innovation, explosive growth, and amazing price/performance improvements.

**Fig. 11:**
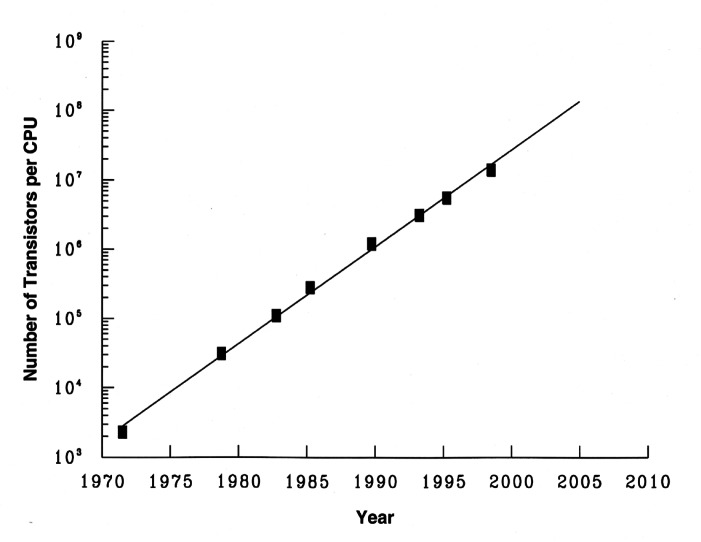
Moore’s Law.

There is one performance forecast that has held up for more than 30 years, Moore’s Law. The observation, made in 1965 by Intel co-founder Gordon Moore, was that each new memory integrated circuit contained roughly twice as much capacity as its predecessor, and each chip was released within 18 to 24 months of the previous chip. This trend of computing power rising exponentially with time has held up well. This law has now been valid for more than three decades, as shown in Fig. 11, and it appears likely to be valid for at least several more device generations.

While computational power has been increasing exponentially, communication speeds have not. This has been due to business factors rather than the lack of technological innovation in tethered and untethered communications. With the recent deregulation of the telecommunications industry, there should be a quantum leap in available communication speeds over the next few years. Competition will drive the increase in speed. New digital wireless systems will lead to the deployment of new mobility products and services. At the end of 2000, about 44.4 million households should be online, compared to about 12.7 million households online in 1995, an increase of nearly 250 % over 5 years. Most of these households will still be connected to the Internet by relatively slow analog modems. Broadband connections such as digital subscriber lines (DSL) and cable modems are now providing speeds that are 10 to 100 times faster than analog modems. At the end of 2000, about 2 million U.S. homes will be connected to the Internet via cable modems and another 1.1 million residential customers will have DSL. The race to provide more economical broadband connections to the household and business is just now starting. The next 10 years should transform our communications infrastructure as we find new ways to exploit advances in computational power.

Requirements for security and interoperability will be top priorities in the information age of the 21st century. As the Internet continues to grow, it will be vulnerable to many threats that can inflict serious damage and cause serious losses. Almost all government and business organizations are dependent on information technology to carry out their activities, from the simplest to the most complex.

Information systems are increasingly open, interconnected and essential to our nation’s commerce, transportation, financial services, communications, and government. These systems can be vulnerable to threats such as accidents, human error, and deliberate attacks unless careful steps are taken to protect them. The threats persist despite efforts to make our information systems more secure and trustworthy and to improve awareness of the serious consequences of poor security.

The use of computers and networks enables U.S. companies to increase productivity and adapt to changing markets. Organizations can provide an integrated collection of low-cost, reliable services and handle tremendous volumes of business and technical transactions. The ability to amass, analyze, and control large quantities of data enables costs to be reduced. While efficiency and accuracy are improved, new ways of doing business and new forms of economic activities will not occur in the absence of continuing improvements in security management and technical methods.

Interoperability will be essential to capture the benefits of new devices that incorporate small computers and sensors. Devices such as televison sets, handheld computers, digital cameras, and laptop computers make it possible to have instant access to information anywhere and at anytime. Forecasts predict that use of these information appliances will outstrip the use of the personal computer by 2002. New applications will be possible only if the devices can interchange information accurately and reliably.

### 5.2 Software

So with the advent of the 21st century, what will be needed in the next 10 years in the way of better measurements for the information age? What is the equivalent to better measurements for electricity for the industrial age of 1900? Specific forecasting for IT is certainly not easy and probably not needed. With increases in computational power, communication speed, connectivity, and multimedia content, IT systems will become increasingly complex. This IT system complexity will be in the hardware, but more critically in the software. When it comes to IT measurements in the next 10 years, measuring software will be the challenge.

The President’s IT Advisory Committee (PITAC) Report, *Investing in Our Future* [61], listed software as its first concern for research:

“Software—The demand for software has grown far faster than our ability to produce it. Furthermore, the Nation needs software that is far more usable, reliable, and powerful than what is being produced today. We have become dangerously dependent on large software systems whose behavior is not well understood and which often fail in unpredicted ways. Therefore, increases in research on software should be given a high priority. Special emphasis should be placed on developing software for managing large amounts of information, for making computers easier to use, for making software easier to create and maintain, and for improving the ways humans interact with computers.”

The report also pointed out the contrasts between software and hardware. During the past 40 years, computing hardware has increased at least eight orders of magnitude in performance. But the ability to develop software has not kept pace. Software is often fragile, unreliable, and extremely difficult and labor-intensive to develop, test, and evolve. Some of the difficulties inherent in software systems pertain to the lack of meaningful and standardized behavioral specifications that would make it possible to test the properties of the systems. Too often software flaws are observable only when the program is run, and even then flaws can be invisible. Often there is no correspondence between a specification and the software itself. Large software systems are complex, unpredictable in performance, and difficult to describe precisely and to test.

IT measurement and testing for software will play a fundamental role in ensuring that complex IT systems are scalable, usable, reliable, secure, and interoperable.

### 5.3 Possible Roles for NIST

After 100 years of service, it is very apparent what essential roles NIST now fulfills in physical and chemical metrology. The importance of those physical and chemical metrology roles is well understood by the metrology community and certainly appreciated by our industry customers. After about 30 years, the roles NIST plays in IT metrology are still evolving. Presently, NIST is valued for specific IT conformance tests, interoperability tests, performance tests, and reference data developed by NIST at the request of government and industry. Over the next 10 years, this role for NIST can be expected to continue. However, advancing the science and technology for software development, especially better methods for developing software tests, may be the most critical need and daunting challenge facing the IT metrology community today.

After a few decades of research and practice, we appear to have achieved a plateau for the state-of-the-art in software testing, a plateau which will not suffice for the large and complex systems of IT systems of next the 10 years. A recent review of the state of software metrics [18] indicated a lack of rigor compared to physical metrology. The physical metrology principles of units, scale, and uncertainty presently have no counterpart in software metrics. At the June 1999 International Conference on Testing Computer Software, the keynote address by Elaine J. Weyuker, *What Has Software Testing Research Done for Me Lately, and What Will It Do For Me in the Future*, reviewed the commercial relevance of recent research. This review included a survey of the four most important journals over five years, from 1994 to 1998. The survey revealed a total of 819 papers, of which only 49 primarily discussed software testing issues. Much of the reported research in those 49 papers was judged by the speaker as not useful to the tester of complex commercial software systems. While software testing as a profession has certainly progressed over the last decade, the software testing researcher has been unable to fulfill the present needs of the software testing practitioner.

What are the consequences of this shortfall in software testing research and consequently software testing practice? NIST is presently attempting to quantify the economic consequences. In 1999, NIST initiated a study on the *Economic Impacts of an Inadequate Infrastructure for Software Testing*. The scope of this study was expanded in 2000 and the final report should be available sometime in 2001. Identifying economic impacts should help to identify and to focus research priorities. As a national metrology institution with expertise in software measurement and testing practice and research, NIST has an excellent window of opportunity over the next ten years to continue to pursue research and development that could significantly advance the state of software testing.

This also may be the opportune time to develop comprehensive reports on current best practices in IT conformance, interoperability, and performance testing. Widely accepted and recognized best practices would assist current IT testing developers and practitioners. Widely accepted and recognized best practices for today, and their limitations, would also help to identify and focus on the research priorities needed to get to the best practices that software testing practitioners will need in 2010. Again, based upon NIST’s IT measuring and testing expertise and role as a national metrology institution, NIST is well positioned to serve as a catalyst and leader in working with industry, government, and academia on these issues.

## 6. Conclusions

Information technology (IT) has transformed the way that people work, learn, and communicate, and contributed significantly to the U.S. economic growth in the 20th century. IT has been the engine for improved productivity, technological innovation, and new opportunities. Today IT is the foundation for many sectors of the American economy, including the transportation, electrical power, banking, water supply, and telecommunications systems that support the basic infrastructures in the United States.

As the use of IT continues to increase, advances in IT metrology will be critical to support the development of systems that are scalable, usable, secure, and interoperable. Users of cell phones, hand-held personal digital assistants, laptop computers and other wireless devices will need devices that are interoperable and capable of securely and reliably exchanging data. People are using mobile cell phones to access the Internet, shop, pay bills, trade stocks, and a host of other applications. The use of mobile cell phones for these electronic commerce applications is expected to rise from about 1 million users in 2000 to about 45 million users in 2004.

People rely more and more on their computers and their wireless devices that provide computing functions. Additionally, information security requirements continue to escalate. New needs are appearing every day. In the 21st century, almost everyone will be dependent on IT systems to perform essential functions. As the technology changes to provide new capabilities and features, new methods for measuring and testing IT will be essential to continued growth and development.

## Footnotes

1 Certain commercial equipment, instruments, or materials are identified in this paper to foster understanding. Such identification does not imply recommendation for endorsement by the National Institute of Standards and Technology, nor does it imply that the materials or equipment identified are necessarily the best available for the purpose.

## Glossary

ADP: Automatic Data Processing
AES: Advanced Encryption Standard
AFIS: Automatic Fingerprint Identification System
AH: Authentication Header
AIAG: Automotive Industry Action Group
ANSI: American National Standards Institute
ANSIDT: American National Standard Dictionary of Information Technology
APs: Application Protocols
ARPA: Advanced Research Projects Agency
ATEP-CMS: Algorithm Testing and Evaluation Program for Coordinate Measuring Systems
ATSs: Abstract Test Suites
BASIC: Beginner’s All-Purpose Symbolic Instruction Code
CAD: Computer-Aided Design
CATIA: Computer Assisted Three-dimensional Interactive Application
CC: Common Criteria
CD-ROM: Compact Disk- Read Only Memory
CEC ERIM: Center for Electronic Commerce at the Environmental Research Institute of Michigan
CIS: Component Interaction Specification
CPU: Central Processing Unit
CMSs: Coordinate Measuring Systems
CMT: Cryptographic Module Testing
CMVP: Cryptographic Module Validation Program
COBOL: Common Business Oriented Language
CORBA: Common Object Request Broker Architecture
CSE: Communications Security Establishment
CSMA/CD: Carrier Sense Multiple Access with Collision Detection
DARPA: Defense Advanced Research Projects Agency
DAT: Digital Audio Tape
DES: Data Encryption Standard
DoD: Department of Defense
DOM: Document Object Model
DSL: Digital Subscriber Lines
DSS: Digital Signature Standard
ESP: Encapsulating Security Payload
FBI: Federal Bureau of Investigation
FDT: Formal Description Technique
FIPS: Federal Information Processing Standard
GOSET: French Operational Group for the Standard for Exchange and Transfer
GPS: Global Positioning System
GSA: General Services Administration
HTML: Hypertext Markup Language
IEC: International Electrotechnical Commission
IEEE: Institute of Electrical and Electronics Engineers
IETF: Internet Engineering Task Force
IKE: Internet Key Exchange
IP: Internet Protocol
IPS: Internet Protocol Suite
Ipsec-WIT: IPsec- Web-based Interoperability Tester
ISO: International Organization for Standardization
IT: Information Technology
ITL: Information Technology Laboratory
IUT: Implementation Under Test
LAN: Local Area Network
MCITT: Manufacturer’s CORBA Interface Testing Toolkit
MEL: Manufacturing Engineering Laboratory
MOSIS: Metal Oxide Semiconductor Implementation System
NBS: National Bureau of Standards
NBSNET: NBS NETwork-deployed in 1979
NIAP: National Information Assurance Partnership
NIST: National Institute of Standards and Technology
NIST Net: NIST Network Emulation Tool
NISTIR: National Institute of Standards and Technology Internal/Interagency Report
NSA: National Security Agency
NVLAP: National Voluntary Laboratory Accreditation Program
OEMs: Original-Equipment Manufacturers
OMB: Office of Management and Budget
OMG: Object Management Group
PCM: Pulse Code Modulation
PDM: Product Data Management
PITAC: President’s IT Advisory Committee
PKI: Public Key Infrastructure
SAs: Security Associations
SEAC: Standards Electronic/Eastern Automatic Computer
SHS: Secure Hash Standard
SI: International System of Units
SQL: Structured Query Language
STEP: Standard for the Exchange of Product Model Data
SWAC: Standards Western Automatic Computer
TIMS: Testability of Interaction-driven Manufacturing Systems
TRAMS: TRAce Measurement System
TREC: Text Retrieval Conferences
TS: Technology Services
USPRO: U.S. Product Data Association
VHS: Video Home System
VIM: The International Vocabulary of Basic and General Terms in Metrology
VLSI: Very Large Scale Integration
W3C: World Wide Web Consortium
WSQ: Wavelet Scalar Quantization
WWW: World Wide Web
XML: Extensible Markup Language
xDSL: (generic) Digital Subscriber Line

## References

[1] American National Standard Dictionary of Information Technology (ANSDIT).

[2] (1993). International Vocabulary of Basic and General Terms in Metrology (the VIM).

[3] ISO/IEC Guide 2: 1996, Standardization and related activities—General vocabulary.

[4] Passaglia E, Beal KA (1999). A Unique Institution, The National Bureau of Standards 1950–1969.

[5] Baird GN (1972). The DoD COBOL Compiler Validation System.

[6] Montanez-Rivera C, Johnson LA (1992). Conformance Test Specifications for COBOL Intrinsic Function Module.

[7] NIST Software Standards Validation Group (1993). 1978 Fortran Compiler Validation System User’s Guide, Version 2.1.

[8] Cugini JV, Bowden JS, Skall MW (1980). NBS Minimal BASIC Test Program-Version 2, User Manual, (Volume 1, Documentation and Volume 2, Source Listings and Sample Output).

[9] Flater D, Gallagher L, Hurwitz S, Sullivan J (1996). User’s Guide for the SQL Test Suite Version 6.0.

[10] Baird GN, Johnson LA (1981). Compiler Validation—An Assessment.

[11] Rosenthal R, Meisner NB

[12] Rosenthal R (1984). Local Area Networks: Baseband Carrier Sense Multiple Access with Collision Detection Access Method and Physical Layer Specification and Link Layer Protocol.

[13] Mink A, Carpenter R, Nacht G, Roberts J (1990). Multiprocessor Performance-Measurement Instrumentation. IEEE Computer.

[14] Mink A, Salamon W, Hollingsworth J, Arunachalam R Performance Measurement using Low Perturbation and High Precision Hardware Assists.

[15] Martin C, Mink A, Salamon W, Indovina M, Couson M Performance Measurement of Remote ATM Cluster.

[16] Mink A, Carpenter R, Courson M Time Synchronized Measurements in Cluster Computing Systems.

[17] Hogan MD (1997). Metrology for Information Technology.

[18] Gray M (1999). Applicability of Metrology to Information Technology. J Res Natl Inst Stand Technol.

[19] ISO/IEC TR13233: 1995 Information technology—Interpretation of accreditation requirements in ISO/IEC Guide 25—Accreditation of Information Technology and Telecommunications testing laboratories for software and protocol testing services.

[20] (1994). ISO 10303-1:1994. Industrial automation systems and integration—Product data representation and exchange—Part 1: Overview and fundamental principles.

[21] Kemmerer S (1999). STEP The Grand Experience.

[22] NIST (1999). Federal Information Processing Standard 46.

[23] NIST (2000). Federal Information Processing Standard 186.

[24] NIST (1995). Federal Information Processing Standard 180.

[25] NIST (1994). Federal Information Processing Standard 140.

[26] (1999). ISO/IEC 15408-3, Information Technology—Security Techniques—Evaluation criteria for IT Security, Parts 1–3.

[27] (2000). Document Object Model (DOM) level 2 Core Specification Version 1.0 W3C Proposed Recommendation.

[28] (2000). Extensible Markup Language (XML) 1.0 (Second Edition), W3C Recommendation.

[29] Linn RJ (1990). Conformance Testing for OSI Protocols. Computer Networks and ISDN Systems.

[30] Rosenthal L, Skall M, Brady M, Kass M, Montanez-Rivera C (1997). Web-Based Conformance Testing For VRML. Standard-View.

[31] NIST Cerberus An IPsec Reference Implementation for Linux.

[32] Kent S, Atkinson R (1998). Security Architecture for the Internet Protocol.

[33] Kent S, Atkinson R (1998). IP Encapsulating Security Payload.

[34] Kent S, Atkinson R (1998). IP Authentication Header.

[35] NIST PlutoPlus: An IKE Reference Implementation for Linux.

[36] Harkins D, Carrel D (1998). The Internet Key Exchange (IKE).

[37] Frechette SP (1997). STEP Implementation: Solid Model Exchange Results in the AutoSTEP Project. J Agility Global Competition.

[38] ISO 10303-203:1994 (1994). Industrial automation systems and integration—Product data representation and exchange—Part 203: Application protocol: Configuration controlled 3D designs of mechanical parts and assemblies.

[39] IEEE Standard Glossary of Software Engineering Terminology.

[40] (1996). Project Brief: Advanced Process Control Framework Initiative.

[41] Bloom HM, Christopher N (1996). A Framework for Distributed and Virtual Discrete Part Manufacturing.

[42] CORBA Basics http://sisyphus.omg.org/gettingstarted/corbafaq.htm.

[43] Flater D (1999). Manufacturer’s CORBA Interface Testing Toolkit: Overview. J Res Natl Inst Stand Technol.

[44] MCITT Web Site (1999). http://www.mel.nist.gov/msidstaff/flater/mcitt/.

[45] (2000). American National Standard for Information Systems, Data Format for the Interchange of Fingerprint, Facial, and Scar Mark and Tattoo (SMT) Information.

[46] IAFIS-IC-0110 (V) WSQ Gray-scale Fingerprint Image Compression Specification.

[47] NIST Special Database 9—NIST 8-bit Gray Scale Images of Mated Fingerprint Card Pairs available from NIST SRD program.

[48] Diaz C, Hopp TH (1995). Evaluation of Software for Coordinate Measuring Systems.

[49] Shakarji CM (1998). Least-Squares Fitting Algorithms of the NIST Algorithm Testing System. J Res Natl Inst Stand Technol.

[50] Martin A, Przybocki M (2000). The NIST 1999 Speaker Recognition Evaluation—An Overview, Digital Signal Processing.

[51] Voorhees EM, Harman DK (2000). The Eighth Text Retrieval Conference (TREC-8).

[52] Nechvatal J (2000). Report on the Development of the Advanced Encryption Standard (AES).

[53] Rukhin A (2000). A Statistical Test Suite for Random and Pseudorandom Number Generators for Cryptographic Applications.

[54] Morris KC, Flater D (1999). Standards-based Software Testing in a Net-Centric World.

[55] Flater D (2000). TIMS Project Description.

[56] Flater D (2000). Specification of Interactions in Integrated Manufacturing Systems.

[57] (1998). Object Management Group PDM Enablers.

[58] (1999). STEP PDM Schema Version 1.1.

[59] Flater D, Morris KC Testability of Product Data Management Interfaces.

[60] Morris KC, Flater D Design of a Flexible, Integrated Testing System for STEP and OMG Standards.

[61] (1999). President’s IT Advisory Committee (PITAC) Report, Investing in Our Future.

